# Supplementary material for: Human Papillomavirus in Sinonasal Squamous Cell Carcinoma: A Systematic Review and Meta-Analysis
Source: Cancers (Basel). 2020 Dec 25;13(1):45. doi: 10.3390/cancers13010045 (PMC7796014; doi:10.3390/cancers13010045)
Supplement: Supplementary file 1 [file cancers-13-00045-s001.pdf]

Supplementary Materials

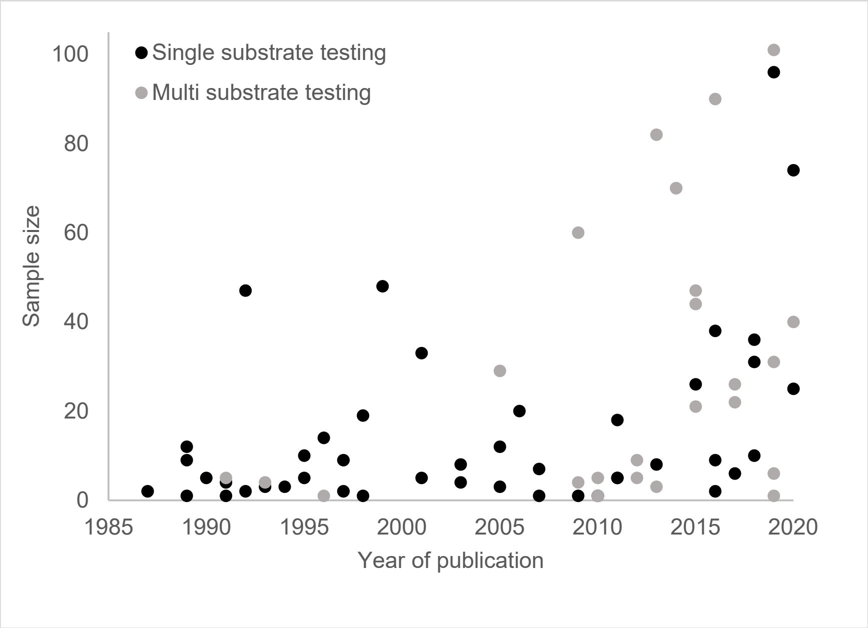

Figure S1 Sample size against year of study publication. Scatterplot depicting sample size against year of study publication for single and multi-substrate testing studies, showing larger and more multi substrate testing studies over the years.

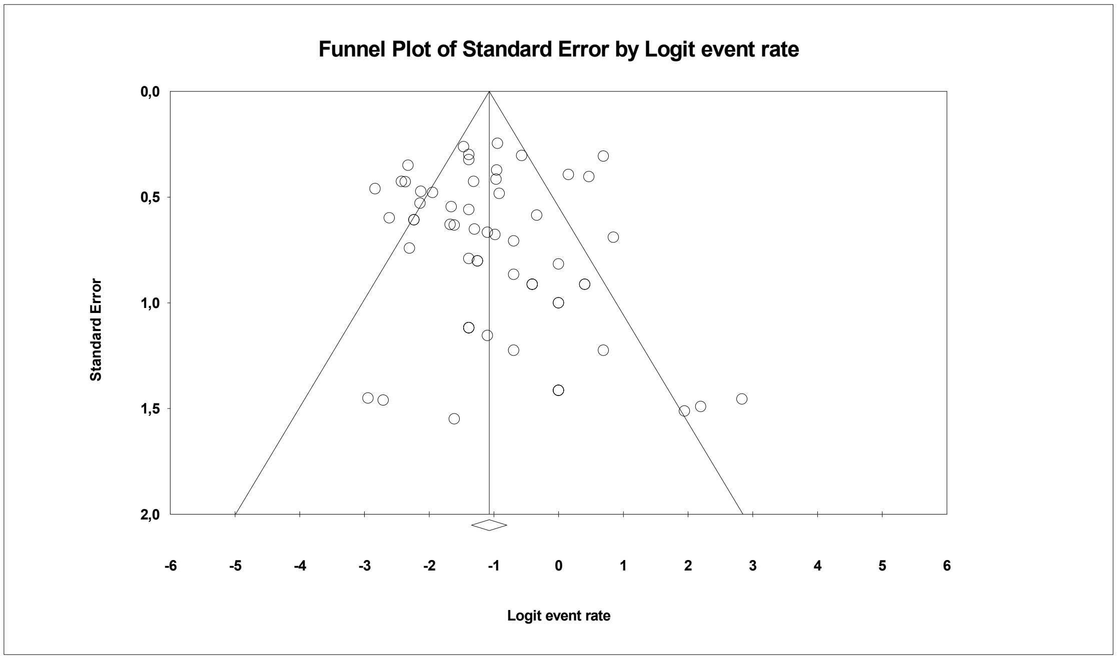

Figure S2 Funnel plot. Funnel plot displaying the standard error by logit event rate for the 61 studies eligible for analysis.

Table S1 Meta regression results. Table showing results from a Random Effects Model to test for association of Detection method, Anatomical Subsite and Geographic Regions with HPV Prevalence.

Variable	Estimate	95% Lower Limit	95% Upper Limit	P-Value	Model Q	P-Value	I^2 (%)	τ^2
**Detection Method**					3.54	0.06	69.5	0.72
Single-agent	Reference							
Multi-agent	0.572	-0.024	1.167	0.06				
**Anatomic Subsite**					6.33	0.04	63.2	0.62
High Exposure Areas	Reference							
Low Exposure Areas	-1.347	-2.442	-0.250	0.02				
Not Specified	-0.579	-1.223	0.066	0.08				
**Geographic Region**								
Europe	Reference				3.12	0.37	68.9	0.66
Africa	-0.820	-2.203	-0.572	0.25				
Asia	-0.164	-0.858	0.530	0.64				
North America	0.268	-0.434	0.971	0.45				

Table S2 Heterogeneity analyses for HPV prevalence subgroups using the fixed-effects model. Heterogeneity analyses of HPV prevalence by detection method (single- vs. multi-agent detection and single- vs. multi-agent with RNA vs. multi-agent without RNA), anatomic subsite, and geographic region.

Subgroup	Fixed-effects analysis	Heterogeneity		Tau-squared			
		Q-value	p-value	Tau-squared	Standard error	Variance	Tau
**Detection method: Single- vs. multi-agent**	Within subgroups	173.76	0.00	-	-	-	-
	Overall	182.75	0.00	0.724	0.245	0.060	0.851
**Detection method: Single- vs. RNA-multi- vs. no-RNA-multi-agent**	Within subgroups	171.24	0.00	-	-	-	-
	Overall	182.75	0.00	0.724	0.245	0.060	0.851
**Anatomic subsite**	Within subgroups	184.97	0.00	-	-	-	-
	Overall	191.82	0.00	0.622	0.197	0.039	0.789
**Geographic region**	Within subgroups	176.55	0.00	-	-	-	-
	Overall	187.60	0.00	0.657	0.216	0.047	0.811

Supplementary Methods

Appendix

A list of sentinel articles was identified before the search was conducted. These sentinel articles (PMID's 23253489, 28271500, 24030745, 26229021, and 23060353) were used to generate search terms and test the effectiveness of the search strategy. The search strategy was peer reviewed by a second medical librarian through the Peer Review of Electronic Search Strategies (PRESS) forum.

Complete PubMed Search

Search 1

Papillomavirus Infections[MeSH] OR Papillomaviridae[MeSH] OR HPV[tw] OR HPV+[tw] OR p16+[tw] OR p16[tw] OR HPV-related[tw] OR alphapapillomavirus*[tw] OR betapapillomavirus*[tw] OR gammapapillomavirus*[tw] OR mupapillomavirus*[tw] OR HPV-16[tw] OR HPV-18[tw] OR papilloma virus*[tw] OR papillomavirus*[tw]   79428 items

Search 2

("DNA Tumor Viruses"[Mesh] OR "Oncogenic Viruses"[Mesh]) AND ("Papillomaviridae"[Mesh] OR HPV[tw] OR HPV+[tw] OR p16+[tw] OR p16[tw] OR HPV-related[tw] OR alphapapillomavirus*[tw] OR betapapillomavirus*[tw] OR gammapapillomavirus*[tw] OR mupapillomavirus*[tw] OR HPV-16[tw] OR HPV-18[tw] OR papilloma virus*[tw] OR papillomavirus*[tw])   32864 items

Search 3

("DNA Virus Infections"[Mesh] OR "Tumor Virus Infections"[Mesh]) AND ("Papillomaviridae"[Mesh] OR HPV[tw] OR HPV+[tw] OR p16+[tw] OR p16[tw] OR HPV-related[tw] OR alphapapillomavirus*[tw] OR betapapillomavirus*[tw] OR gammapapillomavirus*[tw] OR mupapillomavirus*[tw] OR HPV-16[tw] OR HPV-18[tw] OR papilloma virus*[tw] OR papillomavirus*[tw])   30608 items

Search 4

1 or 2 or 3   79428 items

Search 5

sinus*[tw] AND (nasal[tw] OR paranasal[tw] OR ethmoid[tw] OR frontal[tw] OR maxillary[tw] OR sphenoid[tw]) 55236 items

Search 6

sinonasal[tw] OR nasal[tw] OR nose*[tw] 169151 items

Search 7

Paranasal Sinuses [MeSH] OR Paranasal Sinus Neoplasms[MeSH] OR Paranasal sinus diseases[MeSH] OR Nose[MeSH] OR Nose neoplasms[MeSH] OR Nose diseases[MeSH] 146986 items

Search 8

5 or 6 or 7 232640 items

Search 9

“Carcinoma, Squamous Cell"[Mesh] OR "Neoplasms, Squamous Cell"[Mesh] OR “Squamous Cell Carcinoma of Head and Neck”[MeSH] OR Carcinoma*[tw] OR SCC[tw] OR HNSCC[tw] 864305 items

Search 10

4 and 8 and 9 403 items

Search 11

#10 NOT (animals[mh] NOT humans[mh]) 392 items
